# Improved efficacy of an influenza DNA vaccine through high-density microarray patch delivery

**DOI:** 10.1099/jgv.0.002179

**Published:** 2025-11-14

**Authors:** Chloe G. Entriken, Kimberley L. Bruce, Bridget M. Coyne, Saxon H. Kruyer, Jane E. Sinclair, Jovin J.Y. Choo, David A. Muller, Christopher L. D. McMillan

**Affiliations:** 1 School of Chemistry and Molecular Biosciences, The University of Queensland, Brisbane, QLD, 4072, Australia; 2 Australian Infectious Diseases Research Centre, Global Virus Network Centre of Excellence, Brisbane, QLD, 4072, Australia

**Keywords:** influenza, microarray patch, pandemic, vaccine

## Abstract

Pandemic preparedness requires vaccine platforms that are fast to produce, thermostable and suitable for broad deployment. DNA vaccines are well suited to this task but have historically suffered from poor immunogenicity when delivered by conventional intramuscular (IM) injection. Here, we evaluated high-density microarray patch (HD-MAP) delivery of a DNA vaccine encoding the influenza A/California/01/2009 (H1N1pdm09) haemagglutinin (HA) antigen. *In vivo* imaging of a luciferase reporter construct demonstrated earlier and higher expression following HD-MAP application compared to IM injection. HD-MAP delivery of the HA vaccine induced strong HA-specific IgG responses, whereas IM delivery did not. Upon challenge with a homologous H1N1 virus, all HD-MAP-vaccinated mice were protected from weight loss, while 50% of intramuscularly vaccinated mice met humane endpoints. These findings support the use of HD-MAPs to overcome delivery limitations of DNA vaccines and enhance their utility for future outbreak and pandemic response.

**Impact Statement**

DNA vaccines are a promising tool for rapid pandemic response due to their speed of production, stability and low manufacturing cost. However, their clinical performance has been limited, in part due to poor delivery using conventional methods. In this study, we show that high-density microarray patch (HD-MAP) delivery dramatically improves the immunogenicity and protective efficacy of an influenza virus DNA vaccine in mice. This work adds to the growing body of literature showing that HD-MAPs can overcome delivery barriers for multiple vaccine platforms and provides new evidence that they may rescue the performance of DNA vaccines specifically. The technology has broad relevance for global pandemic preparedness, particularly in settings where traditional delivery methods like electroporation are impractical. HD-MAPs are dry-formulated, thermostable and easy to apply, offering needle-free, scalable vaccination without the need for trained healthcare workers. These features make this delivery strategy highly suitable for rapid, equitable deployment in both high- and low-resource settings. This study represents a meaningful step towards real-world use of DNA vaccines, shifting them from a platform of theoretical promise to one with practical utility in future outbreak scenarios.

## Data Summary

The authors confirm all supporting data, code and protocols have been provided within the article.

## Introduction

Influenza remains one of the most significant zoonotic threats to global public health, with a demonstrated capacity to cause pandemics. While seasonal influenza strains impose annual health burdens, novel influenza viruses emerging from animal reservoirs have repeatedly crossed into the human population, leading to widespread morbidity and mortality. The risk of highly pathogenic avian influenza (HPAI) viruses emerging into the population is ever increasing. Since February 2022, the United States has detected HPAI in over 1,000 poultry flocks [1], and infection has been detected in many terrestrial mammals, suggesting adaptation allowing transmission to a wider range of mammalian hosts [2]. Dairy cattle have also been infected, further impacting the agricultural industry [3]. Regions in Southeast Asia have seen a re-emergence of H5N1 infections, stemming from novel reassortant viruses [4].

Vaccination remains the most effective prophylactic measure against influenza. In the event of a pandemic, current vaccine production capacity would likely fall short, taking a long time to manufacture the required doses to protect the population [5]. More recent approaches have utilized mRNA vaccines to achieve good protection against HPAI viruses in preclinical models [6]. While mRNA vaccines can be manufactured rapidly against a novel emerging virus, they face logistical challenges, as they are expensive and often require ultra-low temperature storage, which would limit their equitable deployment during a pandemic [7]. DNA-based vaccines are an alternative approach, as they can also be manufactured rapidly in response to a novel emerging virus. However, DNA vaccines often require large doses or complex electroporation equipment to enhance uptake and efficacy after injection. One approach that has shown promise in overcoming such limitations is the high-density microarray patch (HD-MAP). The HD-MAP is a solid 1 cm^2^ microprojection array containing 5,000 microprojections, each 250 µm in length. The vaccine is dry coated onto the projections before application to the skin at 20 m s^−1^ using a spring-loaded applicator. This delivers vaccine directly to the immune-rich layers of the skin and has shown considerable increases in immunogenicity relative to needle-and-syringe injection for a wide range of vaccine types, including DNA vaccines against SARS-CoV-2 and Zika [8,14]. The HD-MAP has also been used in human subjects, with phase I clinical trials showing efficacy of influenza [15] and measles–rubella [16] vaccines, showing its efficacy is not limited to animal models. Perhaps most importantly, as the HD-MAP microprojections themselves cannot penetrate the skin without an applicator, the vaccines are safe and easy to apply, generating no sharps waste and allowing self-application [15,17]. This makes them ideally suited for rapid rollout to resource-limited settings.

Here, we develop an influenza haemagglutinin (HA)-based DNA vaccine, using the 2009 H1N1 pandemic strain (GenBank accession FJ966082.1) as a model antigen. We demonstrate that HD-MAP delivery results in significantly higher IgG responses compared to standard intramuscular (IM) injection in mice. This immunogenicity translated to complete protection from virus challenge, compared to only 50% protection in the IM-injected mice. This work provides another piece in the toolkit to combat the potential emergence of pandemic influenza viruses in the future.

## Methods

### Cloning and purification of plasmids

The sequence from A/California/1/2009 (H1N1pdm) HA was ordered as a synthetic geneblock from Integrated DNA Technologies. The geneblock contained 5′ and 3′ sequences suitable for InFusion cloning into KpnI-digested pVAX1 plasmid. The geneblock was cloned into the pVAX1 plasmid via InFusion cloning as per the manufacturer’s protocols. Plasmid DNA was extracted from clones using the Promega PureYield midi-prep kit, and sequence confirmation of the insert was obtained by Sanger sequencing using primers flanking the KpnI InFusion site at the Australian Genome Research Facility. Large-scale plasmid DNA was prepared using the Qiagen EndoFree Plasmid Giga Kit as per the manufacturer’s protocols. DNA was quantified using a NanoDrop One Microvolume UV-Vis Spectrophotometer (Thermo Fisher Scientific).

### HD-MAP coating and delivery efficiency measurement

HD-MAPs were plasma-treated using a HPT-200 plasma cleaner (Henniker Plasma) and then coated using a custom nitrogen jet-based drying coating rig. HD-MAPs were coated with 21 µl of a formulation containing a fivefold excess of plasmid DNA in a 1 % w/v methylcellulose formulation, diluted in sterile water. To determine delivery efficiency, five HD-MAPs were coated and delivered to mice. The plasmid remaining on the HD-MAP was eluted by submersion in 200 µl of water in a 24-well tissue culture plate followed by shaking for 30 min at room temperature. DNA concentration was determined by NanoDrop One Microvolume UV-Vis Spectrophotometer (Thermo Fisher Scientific), and the total plasmid DNA removed from the MAPs was calculated.

### *In vivo* imaging studies

All *in vivo* imaging studies were approved by the University of Queensland animal ethics committee (approval number 2021/AE000340). BALB/c mice were purchased from OzGene (Perth, Australia) and housed at the Centre for Advanced Imaging within the University of Queensland. Mice were vaccinated with 25 µg of pVAX1-luciferase either via IM injection or HD-MAP application. On days 1, 2, 3, 4 and 7 post-vaccination, mice were anaesthetized and injected with 200 µl of XenoLight d-Luciferin Potassium salt diluted to 15 mg ml^−1^. After 5 min, luciferase activity was measured on the IVIS Lumina X5 Imaging System.

### Vaccination studies

All animal studies were approved by the University of Queensland animal ethics committee (approval number 2023/AE000467). C57BL/6 mice (*n*=6/group) were purchased from OzGene (Perth, Australia) and housed at the Australian Institute for Bioengineering and Nanotechnology. Mice were immunized with 12.5 µg of pVAX1-HA plasmid either by IM injection or HD-MAP application. Plasmid DNA was formulated in a HD-MAP-coating solution containing 50 µg of plasmid DNA and 1 % w/v methylcellulose diluted in water. Delivery efficiency studies were performed to determine the 20% delivery efficiency value. IM injections were formulated based on this 20% value and diluted further in sterile water prior to injection to ensure dose- and excipient-matched formulations across both vaccination routes. All mice were immunized twice, at 21-day intervals. Blood was taken via retro-orbital bleed on day 20 and via cardiac bleed on day 42. Blood was allowed to clot overnight at 4 °C before centrifugation at 10,000×***g*** at 4 °C for 10 min to collect serum. Samples were stored at −20 °C until analysis.

### Virus challenge studies

C57BL/6 mice were immunized as before via IM injection or HD-MAP application. On day 42 (21 days after the second dose), mice were challenged with 10^2^ p.f.u. of A/Auckland/1/2009 (H1N1pdm09) virus in 50 µl of PBS via intranasal inoculation under isoflurane anaesthesia. Mice were monitored daily for weight loss and sacrificed by carbon dioxide asphyxiation once the weight loss exceeded 15% of their initial body weight.

### Enzyme-linked immunosorbent assay

Influenza HA protein was expressed and purified as previously described [18] and coated at 2 µg ml^−1^ in PBS on Nunc Maxisorp ELISA plates overnight at 4 °C. Plates were blocked with 150 µl per well of blocking buffer [5% milk diluent blocking solution concentrate (Seracare) diluted in PBS with 0.05% Tween-20 (PBST)] for 30 min at room temperature. Serum samples were serially diluted in blocking buffer in a separate round-bottom 96-well plate before being transferred to the ELISA plate. Serum was allowed to bind to the HA for 1 h at 37 °C before washing three times in PBST. Plates were dried, and then 50 µl per well of a secondary antibody solution (goat anti-mouse HRP, 1 : 2000 in blocking buffer) was added. Plates were incubated for 1 h at 37 °C before washing six times in PBST. Fifty microlitres per well of BioFX TMB One component HRP Microwell substrate (Surmodics Inc.) was then added, and colour was allowed to develop for 5 min before the reaction was stopped by the addition of 50 µl per well of 1M H_2_SO_4_. Absorbance was then read at 450 nm on a Multiskan FC Microplate Photometer (Thermo Fisher Scientific).

### Scanning electron microscopy of HD-MAPs

HD-MAPs were coated with 15 nm of platinum and imaged at a 45° angle by scanning electron microscopy using a Hitachi SU3500 at the Centre for Microscopy and Microanalysis at the University of Queensland.

### Immunofluorescence microscopy

Coverslips (12 mm) were flame-dried after methanol coating and placed into 24-well tissue culture plates. Each coverslip was treated with 300 µl poly-l-lysine (0.01 % w/v in PBS) for 30–60 min, then washed twice with 293T growth medium (Dulbecco’s Modified Eagle Medium (DMEM) supplemented with 10% heat-inactivated FBS). 293T cells were seeded at 2×10^5^ cells per well in 0.5 ml growth medium and incubated overnight at 37 °C, 5 % CO₂. Cells were transfected the following day with 500 ng plasmid DNA using Lipofectamine LTX (Thermo Fisher Scientific) according to the manufacturer’s instructions. At 48 h post-transfection, cells were washed twice with PBS, fixed with 4% paraformaldehyde (300 µl per well, 10 min, room temperature) and permeabilized with 0.1% Triton X-100 in PBS (300 µl per well). Blocking was performed with 5% milk diluent (Seracare) in PBS for 30–60 min, followed by incubation with HA-specific recombinant human mAb FI6v3 (25 µg ml^−1^ in blocking buffer, 1 h, room temperature). After three PBS washes, cells were incubated with goat anti-human IgG Alexa Fluor 555 (2 µg ml^−1^) for 1 h in the dark, washed, counterstained with Hoechst (10 µg ml^−1^) and mounted (VECTASHIELD Antifade Mounting Medium). Confocal images were acquired on a Zeiss LSM510 microscope and analysed with ImageJ.

### Flow cytometry

293T cells were seeded in six-well tissue culture plates at a density of 750,000 cells per well in 293T growth medium. As per the manufacturer’s protocols, 2.5 µg of plasmid DNA was transfected using Lipofectamine LTX (Thermo Fisher Scientific), and cells were incubated at 37 °C, 5% CO_2_. Forty-eight hours after transfection, the media was removed, and the cells were washed once with PBS. Cells were blocked with 1 ml of blocking buffer (5% milk diluent blocking solution concentrate (Seracare) diluted in PBS) for 30 min on ice. Media were removed, and the cells were incubated with primary antibody solution (HA-specific mAb 5J8 at 50 µg ml^−1^) or blocking buffer only for unstained controls and incubated for 30 min on ice. Media were removed, and the cells were washed 3× with 1 ml of cold PBS. Cells were then incubated in 1 ml of secondary antibody solution (goat anti-human Alexafluor 647 diluted 1 : 2000 in blocking buffer) or blocking buffer only for unstained controls. Cells were incubated for 30 min on ice, protected from light, before washing thrice with PBS as before. Cells were resuspended in 1 ml of PBS and analysed on a BD Accuri C6 flow cytometer. Data were analysed using FlowJo version 10.10.0.

## Results

**Fig. 1.**
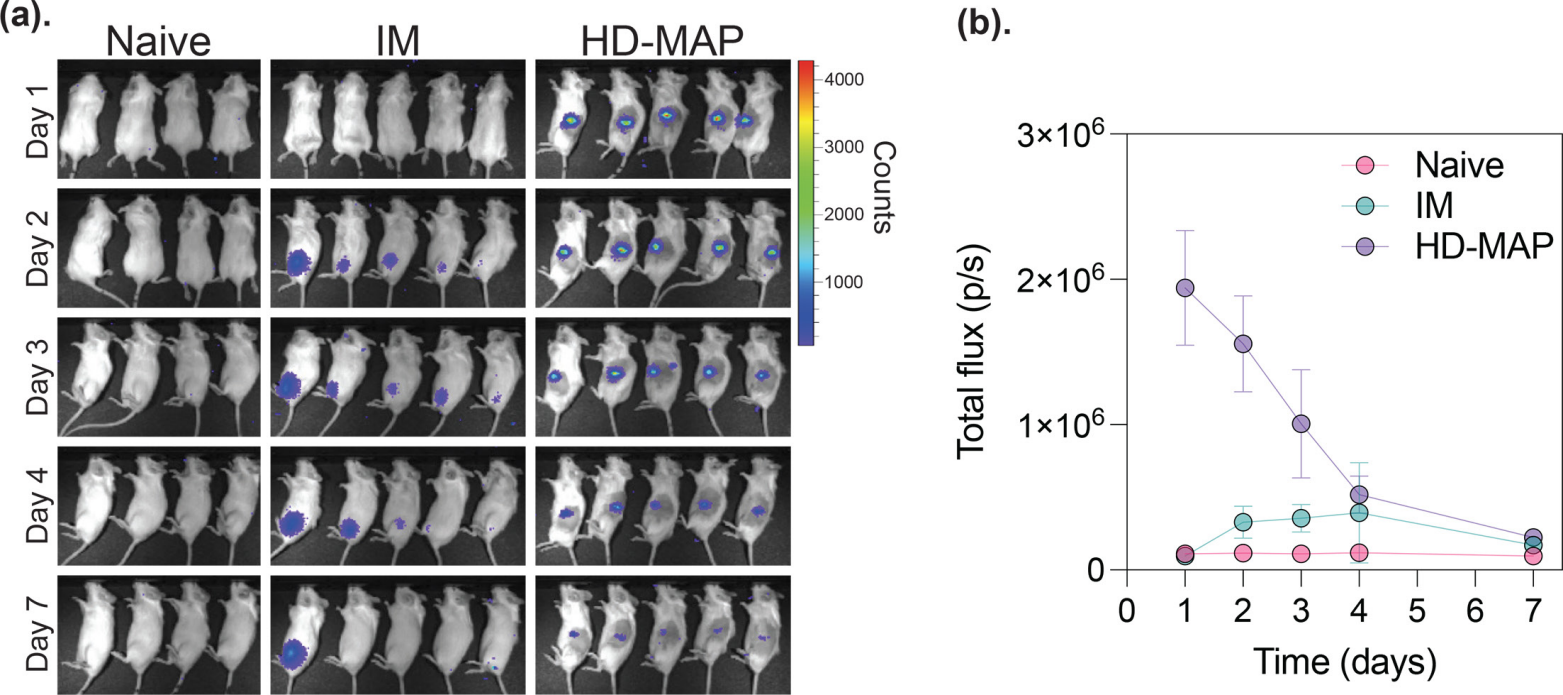
*In vivo* luciferase expression following DNA delivery by HD-MAP or IM injection. BALB/c mice (*n*=4 or 5/group) were vaccinated with 25 µg of pVAX1-luciferase via HD-MAP application or IM injection. (**a**) Representative IVIS images of luciferase expression in each group are shown across days 1, 2, 3, 4 and 7 post-vaccination. (**b**) Quantitation of total luciferase signal (photons/second, total flux) from each mouse over time. Data are presented as mean with error bars representing the sd.

To first determine the expression kinetics of plasmid DNA delivered via the HD-MAP compared to IM injection, we utilized a luciferase reporter construct. BALB/c mice were immunized with 25 µg of the pVAX1-luciferase construct via HD-MAP application or IM injection and assayed on days 1, 2, 3, 4 and 7 for luciferase activity (Fig. 1a). By 1-day post-vaccination, all mice in the HD-MAP group had measurable luciferase expression, whereas those in the IM group only had detectable expression by day 2. Quantitation of the luciferase expression, measured as total flux (p/s), revealed that the HD-MAP mice had almost two logs higher levels on day 1, with expression lowering on days 2 and 3, returning to similar levels as the IM by day 4 (Fig. 1b). By day 7, the majority of expression had returned to baseline levels in all mice.

**Fig. 2.**
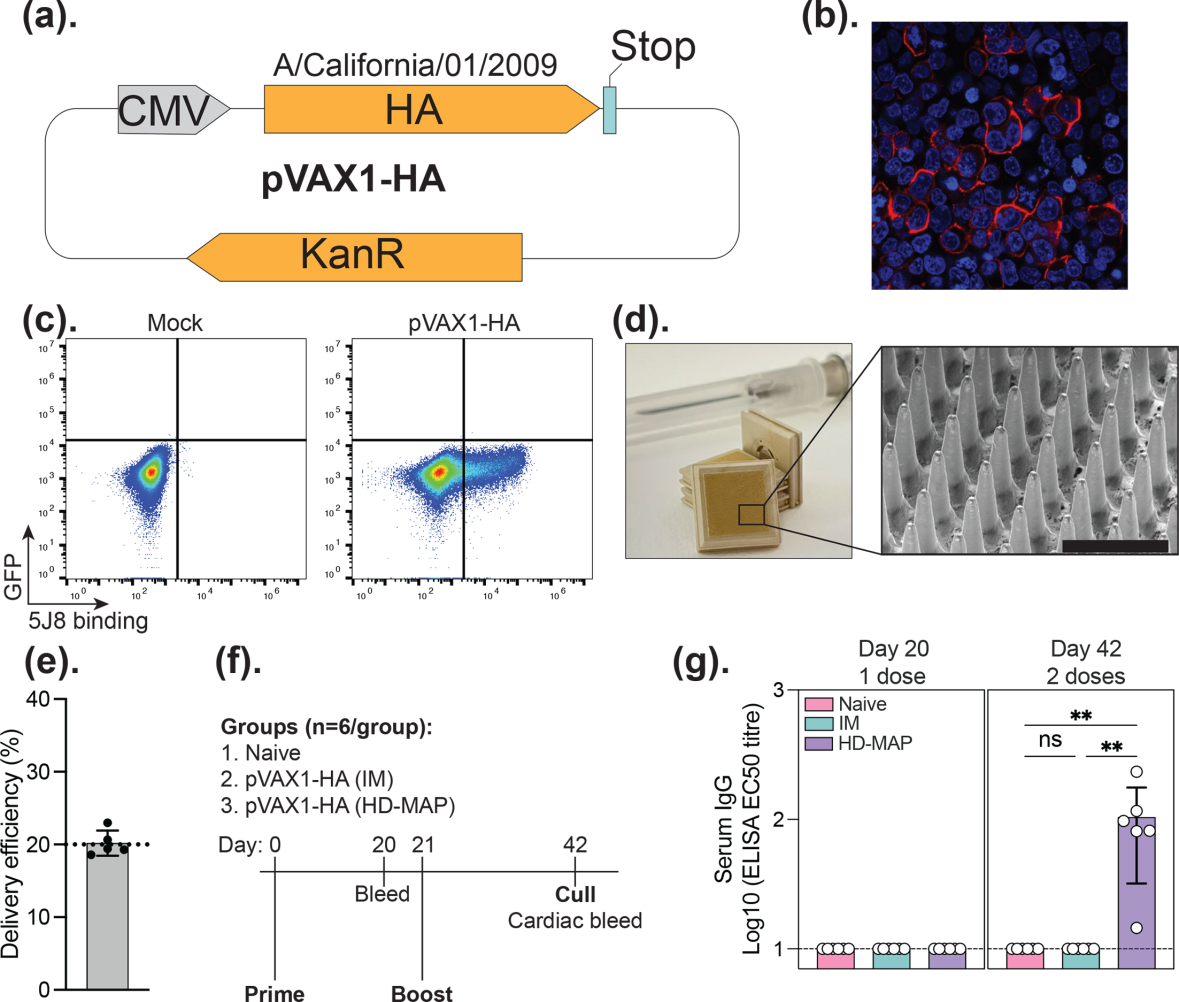
Validation of the pVAX1-HA DNA vaccine construct delivered by the HD-MAP. (**a**) Schematic of the pVAX1-HA plasmid encoding the A/California/01/2009 (H1N1pdm09) HA gene. (**b**) Representative immunofluorescence image showing HA expression on the surface of 293T cells transfected with pVAX1-HA and stained with FI6v3 (red) and Hoechst (blue). (**c**) Flow cytometry analysis of HA expression in 293T cells 48 h post-transfection, using HA-specific antibody 5J8 binding to detect surface HA. (**d**) Photograph of a coated HD-MAP with a 27G needle included for scale, with a scanning electron microscopy image of a DNA-coated HD-MAP. Scale bar is 300 µm. (**e**) Delivery efficiency of plasmid DNA, measured by quantifying DNA removed from the HD-MAP after application to mouse skin (*n*=5). (**f**) Schematic of the immunization schedule used in the mouse study, including two doses administered 21 days apart and sample collection timepoints. (**g**) Serum IgG titres against recombinant HA protein measured by ELISA after one and two vaccine doses delivered via HD-MAP or IM injection. Data represent the mean with error bars representing the sd.

Next, we developed an HA-expression pVAX1 construct (pVAX1-HA), encoding the A/California/01/2009 (H1N1pdm09) HA antigen (GenBank accession FJ966082.1, Fig. 2a). To validate the expression, we performed immunofluorescent microscopy (Fig. 2b) and flow cytometry (Fig. 2c) on transfected cells, using HA-specific mAbs FI6v3 [19] or 5J8 [20]. In both assays, clear expression of HA was observed on the surface of cells. Once the construct was validated, we proceeded to HD-MAP coating and analysed coating morphology by scanning electron microscopy (Fig. 2d). This revealed an even coating on the microprojections with minimal bridging – key characteristics to ensure efficient delivery. Analysing the delivery efficiency revealed 20% of the coated DNA was removed upon application to mouse skin (Fig. 2e). Next, we proceeded to assess the immunogenicity in a mouse model. C57BL/6 mice were immunized in a prime-boost regime, with 21-day intervals between doses (Fig. 2f). ELISA analysis of serum collected on days 21 and 42 revealed induction of high titres of HA-specific IgG in HD-MAP immunized mice, but no detectable IgG in IM-immunized groups (Fig. 2g).

**Fig. 3.**
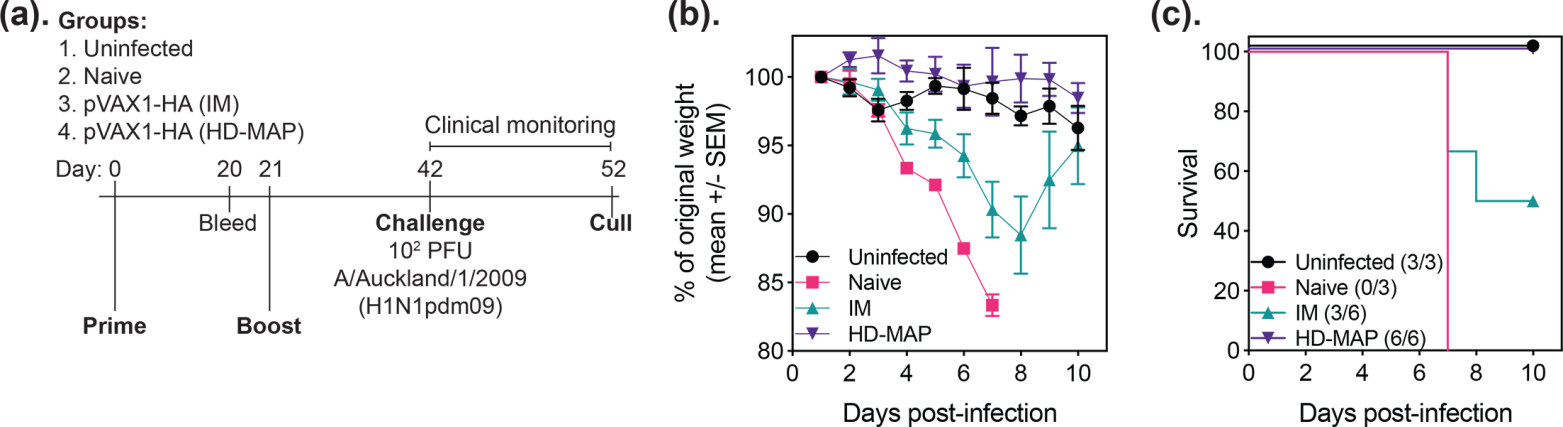
Protection from influenza virus challenge following DNA vaccination via the HD-MAP. (**a**) Schematic of the immunization and challenge timeline. Mice received two doses via HD-MAP or IM injection, 21 days apart, followed by a challenge with the A/Auckland/1/2009 (H1N1pdm09) virus 21 days after the second dose. (**b**) Weight loss over and (**c**) Survival in the 10 days following intranasal virus challenge. Data represent the mean with error bars representing the sem.

Given the promising immunogenicity assays, we next proceeded to a virus challenge study. Mice were immunized as before and challenged on day 42 (21 days after the final immunization) with 10^2^ p.f.u. of the A/Auckland/1/2009 (H1N1pdm09) virus (GISAID isolate ID EPI_ISL_30628) (Fig. 3a). This virus has a matched HA sequence to the vaccine immunogen. Mice were monitored for weight loss for 10 days after infection. All uninfected and HD-MAP-vaccinated mice showed no appreciable weight loss across the course of the study (Fig. 3b). Those unvaccinated or IM-vaccinated mice rapidly lost weight, resulting in all the naïve mice being euthanized by day 7 post-infection (Fig. 3c). Three of the IM-vaccinated mice also fell below the 15% weight loss cutoff and were euthanized, and the remaining three mice began to regain weight and were close to baseline weight at the study end.

## Discussion

The COVID-19 pandemic has renewed global focus on vaccine technologies that are adaptable, rapidly manufactured and suitable for broad deployment. While mRNA platforms have proven transformative, they require specialized infrastructure and cold-chain logistics, severely limiting their efficacy in low-resource settings. DNA vaccines offer several advantages for pandemic response that also address logistical barriers, including speed of design, thermostability and cost-effective manufacturing. However, their utility has been limited by inefficient *in vivo* delivery and weak immunogenicity when administered via standard needle-and-syringe injection, often requiring electroporation after injection. This limitation was highlighted during the pandemic, where leading DNA candidates such as Inovio’s INO-4800 failed to progress beyond early clinical trials despite promising preclinical data [21].

In this study, we show that delivering a DNA vaccine encoding the influenza HA antigen via the HD-MAP markedly improves immunogenicity and protection compared to IM injection. Our results support the idea that the underperformance of DNA vaccine can be overcome by more effective delivery methods.

Using a luciferase reporter system, we first demonstrated that HD-MAP application led to earlier and higher transgene expression *in vivo* compared to IM injection. Expression was detectable by day 1 in all HD-MAP-vaccinated mice, whereas IM delivery showed delayed onset and lower magnitude. This suggests that skin-targeted delivery via HD-MAP enhances cellular uptake and supports more efficient antigen production *in vivo*, at least in the early phase post-vaccination.

Vaccination studies in mice revealed a stark difference in immune responses between delivery methods. HD-MAP immunization induced high titres of HA-specific IgG, detectable after a single dose and further boosted after the second dose. In contrast, IM vaccination with a matched dose and formulation failed to elicit detectable antibody responses. This immunogenicity translated into protection. In a stringent homologous virus challenge model, all HD-MAP-vaccinated mice were protected from weight loss and disease, while 50% of the IM-vaccinated animals required euthanasia. These findings demonstrate that HD-MAP delivery not only improves humoral responses but also confers meaningful protection from viral challenge. Previous DNA vaccination studies used other delivery devices such as the gene gun and showed complete protection with doses less than 1 µg of HA-encoded plasmid DNA [22]. DNA vaccination via injection of chickens (100 µg dose) showed minimal HA-specific IgG titres post-vaccination and boost; however, it still showed partial protection [23]. This aligns with our results, where despite undetectable HA IgG titres by ELISA, we still saw 50% protection in IM mice, likely highlighting the role of T cell immunity in driving this protection.

Although we did not compare HD-MAP delivery to electroporation in this study, electroporation remains the most widely used method for enhancing DNA vaccine uptake in preclinical and clinical settings. While effective, electroporation requires costly, device-specific equipment, trained personnel and mains power, making it poorly suited for large-scale deployment in resource-limited or remote settings. In contrast, HD-MAP delivery is a fully integrated system that requires no external power source and can be applied in seconds. The dry-coated plasmid DNA patches have been shown to be stable at ambient and elevated temperatures [14] and can be self-administered or applied by minimally trained personnel, eliminating the need for medically qualified vaccinators [17]. While the nitrogen jet-based drying process employed here is suitable for research-scale studies, Vaxxas has developed an ‘M-Jet’ printing process that allows vaccine to be coated directly on the tips of the microprojects. This improves vaccine delivery efficiency and is being developed for at-scale manufacture to facilitate transition from preclinical to late-stage clinical studies and beyond [16]. These practical advantages, combined with the improved immunogenicity demonstrated here, make HD-MAPs a compelling alternative to electroporation for DNA vaccine delivery in outbreak and pandemic contexts. It is worth noting that DNA vaccines have shown promising preclinical animal data in the past and then failed to replicate that data in human subjects [21]. Therefore, while this HD-MAP vaccine data are promising, determining whether the efficacy translates to humans is required. Nonetheless, while DNA vaccines alone may not compete directly with mRNA platforms in terms of peak efficacy, their combination with efficient delivery systems like HD-MAPs offers a compelling strategy worth investigating in future studies to diversify our vaccine toolkit.

Further studies could explore the durability of the immune response, the breadth of protection across influenza strains and the cellular immune responses induced. Computational tools to optimize the expression of the antigen could also be applied to further improve efficacy [24]. Nevertheless, this work provides a proof-of-concept that HD-MAP delivery can overcome a key barrier to DNA vaccine performance and supports continued investment in this platform as part of a broader pandemic response strategy.

## Abbreviations

DMEM: Dulbecco's Modified Eagle Medium
HA: haemagglutinin
HD-MAP: high-density microarray patch
HPAI: highly pathogenic avian influenza
HPAI: highly pathogenic avian influenza
IM: intramuscular
mAb: monoclonal antibody
PBST: PBS with 0.05% Tween-20
